# Quality of life, healthcare usage and finances of UK cancer survivors five years post-diagnosis: a matched controlled study

**DOI:** 10.1007/s11764-024-01708-x

**Published:** 2024-12-03

**Authors:** Lorraine Warrington, Kate Absolom, Paul Baxter, Chris Bojke, Gemma Clarke, Samantha Crossfield, Colin Johnston, Adam Martin, Ciaran D. McInerney, Gwen Saalmink, Michele Siciliano, Elizabeth Stamp, Galina Velikova, David Wilkinson, Barbara Woroncow, Penny Wright, Kieran Zucker, Geoff Hall, Adam Glaser

**Affiliations:** 1 Patient Centred Outcomes Research, Leeds Institute of Medical Research at St James’s, University of Leeds, Level 6 Bexley Wing, Leeds, UK; 2 Leeds Institute of Cardiovascular and Metabolic Medicine, School of Medicine, University of Leeds, Leeds, UK; 3 Academic Unit of Health Economics, Leeds Institute for Health Sciences, School of Medicine, University of Leeds, Leeds, UK; 4 Academic Unit of Palliative Care, University of Leeds School of Medicine, Leeds, UK; 5 Leeds Institute of Data Analytics, University of Leeds, Leeds, UK; 6 School of Medicine & Population Health, University of Sheffield, Sheffield, UK; 7 Leeds Teaching Hospitals NHS Trust, Leeds, UK; 8 Department of Health Sciences, University of York, York, UK; 9 School of Sport, Exercise, and Health Sciences, Loughborough University, Loughborough, UK; 10 PPI Member, Leeds Institute of Medical Research at St James’s, University of Leeds, Leeds, UK

**Keywords:** Cancer survivors, Quality of life, Matched controls, PROMs

## Abstract

**Purpose:**

Assessing the long-term impact of cancer on people’s lives is challenging due to confounding issues such as aging and comorbidities. We aimed to investigate this impact by comparing the outcomes of cancer survivors with a matched control cohort.

**Methods:**

This was a cross-sectional survey of breast, colorectal and ovarian cancer survivors approximately 5 years post-diagnosis and a cohort of age, sex and social deprivation-matched controls who had never had a cancer diagnosis. Eligible participants were invited by post to complete a survey assessing quality of life (QoL), health, identity, healthcare usage and finances.

**Results:**

A total of 2075 out of 5734 (36.2%) eligible participants participated (852 cancer survivors and 1223 matched controls). Cancer survivors had poorer QoL than matched controls as assessed by the Quality of Life of Adult Cancer Survivors (QLACS) summary score (*p* = 0.007); however, the effect size was modest (*ω*^2^ = 0.121). The cancer survivors also reported worse outcomes across some individual domains of QoL and health, but not others, and differences were small. There were few differences between cohorts across healthcare usage and finances.

**Conclusions:**

Five years or more after diagnosis, the QoL, healthcare usage and finances of breast, colorectal and ovarian cancer survivors were generally similar to that of age, sex and IMD-matched controls.

**Implications for Cancer Survivors:**

This finding has important implications for people affected by cancer and those providing care who would benefit from greater information on outcomes and functioning beyond treatment. Despite this reassuring finding, it is important to note that there were some differences, on both physical and psychosocial issues, mandating the need for specialist service provision.

**Supplementary Information:**

The online version contains supplementary material available at 10.1007/s11764-024-01708-x.

## Introduction

An estimated 3 million people are currently living with or beyond a diagnosis of cancer in the UK. This is expected to grow to 5.3 million by 2040, due primarily to increases in survival arising from better diagnosis and treatment [1]. Over half of people diagnosed with cancer now survive for over 10 years [2].

Ongoing issues relating to cancer and treatment can affect people’s quality of life (QoL) many years after diagnosis [3]. These may be physical such as pain, fatigue, urinary or bowel incontinence, mobility issues or psychosocial issues including anxiety, depression, adjustment disorders, sexual problems, relationship issues and financial concerns [4–6]. Longitudinal studies show risk factors associated with poorer QoL amongst adult cancer survivors include younger age, lower socio-economic status, unemployment, economic inactivity, comorbidities, low self-efficacy and lack of social support [7–9].

Existing studies comparing QoL amongst cancer survivors to people with no history of cancer, matched for characteristics such as age and sex, show mixed results. Higher rates of anxiety, depression, pain, fatigue, sleep problems and sexual dysfunction have been found amongst breast cancer survivors compared with matched controls up to 10 years after diagnosis [10–12]. However, some positive differences have also been found such as higher levels of post-traumatic growth and greater social support [13, 14]. Evidence suggests that the magnitude of differences in QoL between cancer survivors and matched controls may decrease over time across certain domains [15, 16]. However, limitations of existing studies include small sample sizes, with most studies focusing on one specific group of patients a short time after diagnosis.

One challenge in determining which physical or psychosocial issues are directly attributable to cancer and its treatment, rather than natural aging or comorbidities, is a shortage of matched controlled studies utilising patient-reported outcome measures (PROMs) data.

The work described in this paper was part of a larger project to create a clearer picture of the impact of cancer 5 years after a diagnosis [17, 18].

## Aims

We aimed to investigate the long-term, wider impacts of a breast, colorectal and ovarian cancer diagnosis on overall QoL, psychological, financial and social aspects of people’s lives. The objective was to compare outcomes for a cohort of cancer survivors (breast, colorectal and ovarian) with a matched group of individuals without cancer.

### Outcomes

The primary outcome was QoL measured by the Quality of Life in Adult Cancer Survivors (QLACS) scale [19]. Secondary outcomes were health-related QoL (assessed by symptom items from the EORTC item library [20] and the EQ-5D-3L and Visual Analogue Scores (VAS)[21]), cancer survivor identity, healthcare usage and personal (and household) finances.

## Methods

### Study design

The full study protocol has been published elsewhere [17]. In summary, cross-sectional PROMs data was collected using a survey of breast, colorectal and ovarian cancer survivors 5 years post-diagnosis and a cohort of controls matched for age, sex and Index of Multiple Deprivation (IMD) who had never been diagnosed with cancer.

### Eligibility criteria

All eligible participants were adults aged 18–100 years who were (i.) registered on the Leeds Teaching Hospitals NHS Trust (LTHT) electronic patient record (EPR); (ii.) listed as being registered with a Leeds Clinical Commissioning Group primary care practice; and (iii.) not opted out of research participation nationally or locally.

Eligible participants for the cancer survivor cohort were approximately 5 years beyond an initial diagnosis of breast, colorectal or ovarian cancer, selected based on cancer diagnosis between January 2008 and end July 2015 inclusive. Eligible participants for the matched control cohort were selected from patients reviewed by LTHT dermatology services on a 2-week wait (2WW) for possible skin cancer between December 2006 and end December 2016 inclusive, removing any patient with a subsequent dermatology appointment within a year, or any patient with a diagnosis of cancer previously or subsequently.

This group was deemed to be most representative of the general healthy population, from those cohorts which could be identified from hospital records.

### Study processes

#### Matching

Matching was done on a 2:1 ratio of control cases to cancer cases. For each cancer patient, two matching control patients were randomly selected from the remaining control pool with the same sex (as recorded in the EPR), same IMD quintile group, birth date within 30 months and appointment within 12 months of cancer diagnosis.

For analysis purposes, the first two letters of each participant’s IDs enabled us to identify which participants were cancer survivors and from which disease group, and which were matched controls. For individual cancer group comparisons, we selected control groups based on age, sex and IMD using the SPSS matching function.

#### Recruitment and data collection

We ran a computerised query on the LTHT EPR database, based on eligibility criteria to identify 6000 eligible participants. Full details of the processes used to manage patient identification and invitations to participate are described in full elsewhere [17]. Eligible participants (cancer cases and controls) were posted a letter and participant information sheet describing the study and details about how to log on to the secure online system QTool, sign a digital consent form and complete the survey. A paper version of the consent form and survey, along with a freepost envelope for return, were also provided. Those who declined via phone, email or returning the blank questionnaire were classed as active decliners. Those who did not respond via any medium following a reminder letter sent out 4 weeks later were classed as passive decliners.

#### Data linkage

Survey data was linked to patient-level clinical data extracted from the LTHT EPR via a process of double pseudonymisation [18]. This enabled privacy-preserving integration of additional structured data from routine clinical records such as age, sex and IMD.

### Survey design

The PROMs survey was developed for the cancer and control groups with extensive involvement from clinicians and patient representatives [17]. An overview of PROMs included in the survey is outlined in Table 1.

**Table 1:** Overview of PROMs included in the survey

Name	Concept	Scoring and ranges
**QLACS **[19]	• Designed to assess QoL in adult cancer survivors and can be adapted to assess QoL in non-cancer populations for comparison [19] • To adapt the QLACS for the matched control group, items from the domains *benefits of cancer* and *distress about recurrence* were omitted • Wording of items in the cancer-specific domains which specifically referenced cancer or cancer treatment was changed to ‘health’ or ‘healthcare treatment’	• 47 items scored from 1 (never) to 7 (always) • 12 domain subscales (range 3 to 18) consisting of 3–4 items per domain • 7 generic domains (*negative feelings, positive feelings, cognitive problems, pain, sexual function/interest, energy/fatigue* and *avoidance*) • 5 cancer-specific domains (*financial problems, benefits of cancer, distress about family cancer, distress about recurrence* and *appearance concerns*) • *Generic summary score* calculated from generic domain scores (range 28–196) • *Cancer-specific summary score* calculated from cancer-specific domain scores (range 19–133) • Higher scores indicate lower/worse quality of life
***EORTC Library items ***[20]	• Designed to assess specific symptoms • Nine symptom subscales from the EORTC item library [22], consisting of 14 items in total, were included. Subscales were tingling and numbness, muscular pain, urinary frequency, urinary incontinence, urinary symptoms, gastrointestinal symptoms, diarrhoea, constipation and abdominal/GI symptoms	• Items are rated from 1 (not at all) to 4 (very much) • Subscale scores are calculated according to the EORTC instructions (range 0–100) • Higher scores indicate worse symptoms • Justification for the selection of these specific symptom subscales is outlined in the protocol paper [17]
**EQ-5D-3L **[21]	• Designed to assess health status across five domains (*mobility, self-care, usual activities, pain* and *anxiety/depression*) • A visual analogue scale (VAS) assesses the overall health state • Generic measure used extensively in economic evaluation	• Each health domain is rated from 1 (no problems) to 3 (severe problems) • Moderate/severe ratings (2/3) were amalgamated to create a binary score on each domain (any problems/no problems), based on previous research and to facilitate comparisons [23–25] • An overall utility score (ranging from − 0.56 to 1) was calculated using standard methods, with a higher score representing better health-related quality of life with 1 being equivalent to perfect health and 0 equivalent to quality of life associated with death. Scores below 0 are possible and reflect states worse than death [26] • VAS is rated 0–100 with a higher score indicating better health [27]
**Cancer survivor identity **[28]	• Single question designed to assess cancer-related identity. Cancer survivors are asked to select a self-description from terms ‘A cancer survivor’, ‘A person who has had cancer’, ‘A cancer patient’, ‘A victim of cancer’ or ‘Other’ • The matched control group was asked to select a description of how they would perceive someone who has had a diagnosis of cancer	• Data is categorical with no hierarchical scoring system
**Healthcare usage and personal finances **[29, 30]	• The CCCQ comprises two subscales (*Communication* and *Navigation*) assessing patient’s experience of cancer care co-ordination • *Communication* items were deemed inappropriate for a cancer survivor population not in active treatment and only *Navigation* subscale (seven items) was included • A financial costs questionnaire was based on a previous study [29] and assessed personal income (employment and social security), informal care and support received, out-of-pocket expenses and use of health or care services including hospital, community healthcare or charity services	• CCCQ items rated from 1 (never) to 5 (always) • *Navigation* subscale has a possible range of 7–35, with higher scores indicating more problems • The financial costs questionnaire comprised descriptive data which was compared between the cancer survivors and matched controls

### Missing data

Within the returned questionnaires, rates of missing data for individual questions were 0.9–2.8% for sociodemographic data, 1.3–2.6% for each component of the EQ-5D-3L and 1.5–2.6% for EORTC items.

Rates of missing data were higher for some items of the QLACS, ranging from 0.9 to 12.0%. Items from the ‘sexual interest and function’ and the ‘new relationships’ question from the social avoidance domain were most affected (6.6–12.0%). In line with previous research, domain scores were classed as missing if two or more items were missing [11]. Where only one domain item was missing, it was replaced by the mean of the patient’s other domain scores. This method was applied to the generic and cancer-specific QLACS summary scores.

### Analysis

Statistical analysis was performed in SPSS version 23. Comparisons between cancer and matched control groups were conducted using parametric tests (e.g. *t*-test, ANOVA) where data met appropriate assumptions. Effect sizes were assessed using Cohen’s *D* where appropriate. Non-parametric tests (e.g. Mann Whitney *U*, chi square) were used where assumptions of data were not met. Due to the number of comparisons between groups, we set the threshold for statistical significance at *p* < 0.01 to reduce the risk of type I error.

## Results

### Recruitment (Fig. 1)

**Fig. 1:**
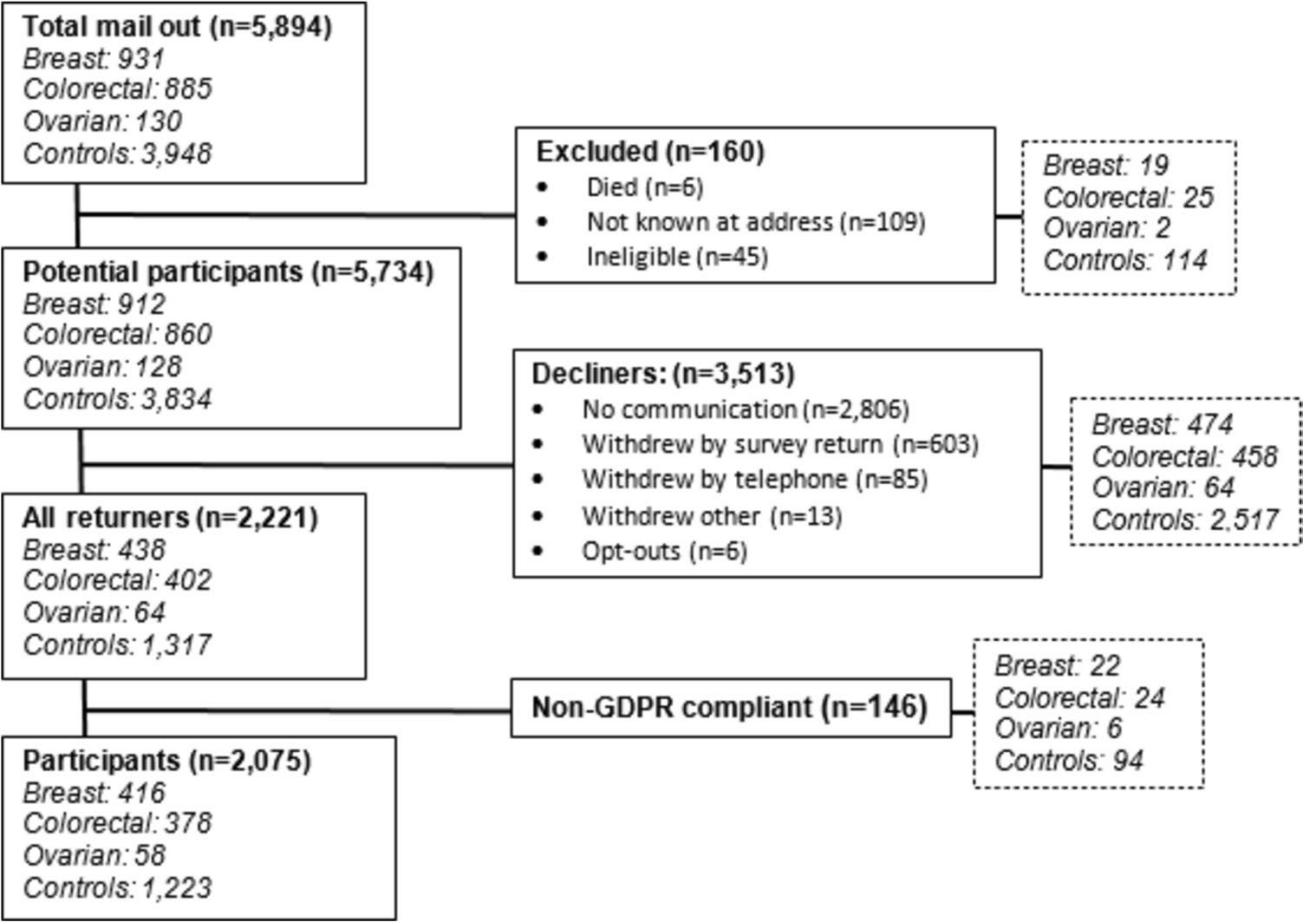
CONSORT diagram of the recruitment process

A total of 5894 potential participants (1946 cancer cases and 3948 controls) were initially invited by mail. One hundred sixty were excluded as ineligible and 3513 declined (2806 passively declined and 707 actively declined). A total of 2221 (38.7%) participants returned completed surveys. However, 146 did not sign the consent form and were excluded from the final sample to ensure compliance with the General Data Protection Regulation. This resulted in a final sample of 2075 respondents (36.2% return rate). Return rates for cancer groups were breast (45.6%), colorectal (42.7%), ovarian (45.3%) and 31.9% for the matched control group.

We compared the 2075 participants to 3507 decliners (3513 minus 6 opt-outs) across age, sex and social deprivation index scores. There were no differences by age or sex, but both the cancer survivors and matched controls had higher levels of participation in the least socially deprived groups compared to the most deprived groups (*p* < 0.001).

### Demographic and clinical data

Table 2 shows the sex, age, IMD and comorbidity characteristics of the overall cancer survivor and the matched control groups, in addition to each subgroup (breast, ovarian and colorectal cancer survivors and their individual control groups). As expected, due to the matching process, the groups were similar in sex, age and IMD profiles and also reported a similar number of comorbidities.

**Table 2:** Proportions of sex, age ranges, IMD and no of comorbidities across the cancer survivor and matched control groups

	All cancer survivors		All matched controls		Breast survivors		Matched controls		Colorectal survivors		Matched controls		Ovarian survivors		Matched controls	
	*N*	%	*N*	%	*N*	%	*N*	%	*N*	%	*N*	%	*N*	%	*N*	%
**Sex**																
Female	637	74.8%	900	73.6%	416	100%	409	100%	163	43.1%	160	43.0%	58	100%	58	100%
Male	215	25.2%	323	26.4%	-	-	-	-	215	56.9%	212	57.0%	-	-	-	-
**Age range**																
25–39	16	1.9%	19	1.6%	12	2.9%	12	2.9%	2	0.5%	1	0.3%	2	3.4%	2	3.4%
40–49	53	6.3%	62	5.0%	37	8.9%	36	8.8%	13	3.4%	11	3.0%	3	5.2%	2	3.4%
50–59	159	18.7%	205	16.8%	116	28.0%	114	27.9%	33	8.7%	42	11.3%	10	17.2%	6	10.3%
60–69	247	29.0%	393	32.2%	133	32.0%	131	32.0%	95	25.1%	94	25.3%	19	32.8%	24	41.4%
70–79	246	28.9%	363	29.7%	86	20.7%	85	20.8%	143	37.8%	144	38.7%	17	29.3%	16	27.6%
80 +	130	15.3%	181	14.8%	31	7.5%	31	7.6%	92	24.3%	80	21.5%	7	12.1%	8	13.8%
**IMD (1, most deprived; 5, least deprived)**																
1	141	16.8%	217	17.8%	74	18.1%	74	18.1%	63	16.8%	65	17.5%	4	6.9%	4	6.9%
2	131	15.6%	166	13.6%	73	17.8%	75	18.3%	52	13.9%	54	14.5%	6	10.3%	12	20.7%
3	160	19.0%	217	17.8%	75	18.3%	74	18.1%	73	19.5%	71	19.1%	12	20.7%	6	10.3%
4	209	24.9%	347	28.5%	97	23.7%	96	23.5%	93	24.9%	103	27.7%	19	32.8%	17	29.3%
5	200	23.8%	270	22.2%	90	22.0%	90	22.0%	93	24.9%	79	21.2%	17	29.3%	19	32.8%
**Comorbidities (number of)**																
0	335	39.3%	449	36.7%	195	46.9%	177	43.3%	114	30.2%	114	30.6%	26	44.8%	21	36.2%
1	279	32.7%	415	33.9%	133	32.0%	119	29.1%	123	32.5%	141	37.9%	23	39.7%	16	27.6%
2	145	17.0%	220	18.0%	57	13.7%	76	18.6%	82	21.7%	70	18.8%	6	10.3%	13	22.4%
3	54	6.3%	88	7.2%	20	4.8%	25	6.1%	32	8.5%	29	7.8%	2	3.4%	6	10.3%
4 +	39	4.6%	51	4.2%	11	2.6%	12	2.9%	27	7.1%	18	4.8%	1	1.7%	2	3.4%

### QLACS

#### Generic summary scores

*T*-test comparisons of the QLACS *generic summary scores* demonstrated that the total cohort of cancer survivors scored higher (*M* = 74, SD = 28.5) than the matched controls (*M* = 70.6, SD = 37.5), indicating worse QoL (*p* = 0.007), although the effect size was modest (*ω*^2^ = 0.121).

Comparisons between individual cancer survivor groups and matched controls identified that although all cancer survivor groups had higher (worse) scores, none of these was statistically significant.

#### Domain scores (Table 3)

**Table 3:** QLACS individual domain scores split by cancer survivors and matched controls

	Cancer survivors			Matched controls			Mann Whitney *U*
	*N*	Mean (SD)	Median (IQR)	*N*	Mean (SD)	Median (IQR)	*p*
**QLACS domains**							
**Energy/fatigue**	836	12.5 (5.4)	12.0 (8.0)	1201	11.7 (5.1)	11.0 (7.0)	0.002*
**Cognitive problems**	842	9.3 (4.6)	8.0 (6.0)	1206	8.9 (4.5)	8.0 (5.0)	0.208
**Positive feelings**	836	20.7 (5.8)	22.0 (10.0)	1203	21.0 (5.5)	22.0 (8.0)	0.137
**Negative feelings**	836	10.3 (4.9)	9.0 (6.0)	1203	10.0 (4.7)	9.0 (6.0)	0.231
**Financial problems**	843	6.9 (4.8)	4.0 (4.0)	1203	5.0 (2.5)	4.0 (0.0)	< 0.001*
**Distress about family cancer**	838	8.3 (5.1)	7.0 (7.0)	1202	5.8 (3.8)	5.0 (4.0)	< 0.001*
**Concerns about appearance**	838	8.0 (5.4)	6.0 (6.0)	1197	5.8 (3.8)	4.0 (2.0)	< 0.001*
**Sexual interest and function**	755	12.1 (6.4)	11.0 (10.0)	1099	10.7 (5.8)	10.0 (9.0)	< 0.001*
**Pain**	841	10.2 (5.8)	8.0 (7.0)	1207	10.3 (5.9)	8.0 (7.0)	0.697
**Social avoidance**	836	8.4 (5.2)	7.0 (6.67)	1200	7.8 (4.9)	6.0 (6.0)	0.004*
**Distress about recurrence**	829	12.0 (6.6)	-	-	-	-	-
**Benefits**	829	16.7 (6.6)	-	-	-	-	-
**EORTC symptoms subscales**							
**Tingling/numbness**	778	20.2 (28.1)	0.00 (33.3)	1145	16.4 (26.2)	0.0 (33.3)	0.001*
**Muscular pain**	778	43.6 (29.1)	33.3 (33.3)	1145	43.8 (29.4)	33.3 (33.3)	0.807
**Urinary frequency**	778	37.7 (27.3)	33.3 (33.3)	1145	36.6 (24.9)	33.3 (33.3)	0.035
**Urinary incontinence**	778	16.7 (25.9)	0.0 (33.3)	1145	14.9 (22.8)	0.0 (33.3)	0.478
**Urinary symptoms**	778	10.6 (22.8)	0.0 (0.0)	1145	7.2 (19.3)	0.0 (0.0)	< 0.001*
**GI symptoms**	778	16.8 (25.3)	0.0 (33.3)	1145	18.5 (25.4)	0.0 (33.3)	0.097
**Diarrhoea**	778	14.4 (24.9)	0.0 (33.3)	1145	9.8 (24.9)	0.0 (0.0)	< 0.001*
**Constipation**	778	18.5 (27.5)	0.0 (33.3)	1145	15.3 (24.3)	0.00 (33.3)	0.038
**Abdominal/GI symptoms**	778	16.7 (18.5)	13.3 (26.7)	1145	13.9 (16.5)	6.7 (20.0)	< 0.001*

*p<.01

Comparisons between all cancer survivors and matched controls across all QLACS domains, excluding the cancer-specific domains (e.g. *distress about recurrence* and *benefits of cancer)*, showed that cancer survivors scored higher (worse) across *energy/fatigue* (*p* = 0.002), *financial problems* (*p* < 0.001), *distress about family cancer* (*p* < 0.001), *concerns about appearance* (*p* < 0.001), s*exual interest/function* (*p* < 0.001) and *social avoidance* (*p* = 0.004). There were no significant differences across the other domains.

In comparing individual cancer groups to their matched controls (Supplementary file/Table 4), we found statistically significant differences between the breast survivors and matched controls across *energy/fatigue* (*p* = 0.004), *financial problems* (*p* < 0.001), *distress about family cancer* (*p* < 0.001), *concerns about appearance* (*p* < 0.001), *sexual interest/function* (*p* = 0.002) and *social avoidance* (*p* = 0.004). There were significant differences between the colorectal group and matched controls across *financial problems* (*p* < 0.001), *distress about family cancer* (*p* < 0.001) and *concerns about appearance* (*p* < 0.001). There was a significant difference between ovarian survivors and matched controls on *financial problems* (*p* = 0.001). There were no statistically significant differences between groups on any of the other domains of QLACS.

#### Cancer-specific summary score

The three cancer survivor groups were compared on the *cancer-specific summary score* of the QLACS using a Kruskal–Wallis test. Statistically significant differences were identified (*p* < 0.001), with the breast group scoring highest (indicating worse QoL), followed by the ovarian group and then the colorectal. Subsequent pairwise analysis using Mann Whitney *U* revealed the differences were between the breast and colorectal groups (*p* < 0.001) only.

### EORTC symptom subscales (Table 3)

Comparisons of the symptom subscale scores of the cancer survivors with their matched controls using Mann Whitney *U* tests found no significant differences across groups on *muscular pain*, *urinary frequency*, *urinary incontinence*, *GI symptoms* or *constipation*. The cancer survivor group reported significantly worse (higher) scores than the matched controls on *tingling and numbness* (*p* = 0.001), *urinary symptoms* (*p* < 0.001), *diarrhoea* (*p* < 0.001) and *abdominal/GI symptoms* (*p* < 0.001).

Comparison of the individual cancer groups (breast, colorectal and ovarian) and their specific matched control groups identified worse *tingling and numbness* (*p* = 0.009) amongst the breast cancer cohort versus their matched controls, but no differences on any other symptoms. The colorectal survivors scored worse than their matched control counterparts on *urinary symptoms* (*p* < 0.001), *diarrhoea* (*p* < 0.001) and *abdominal/GI symptoms* (*p* < 0.001). There were no significant differences on any of the symptom subscales between the ovarian cancer survivors and their matched controls. The full scores are outlined in the Supplementary file/Table 4.

### EQ-5D-3L (Fig. 2)

**Fig. 2:**
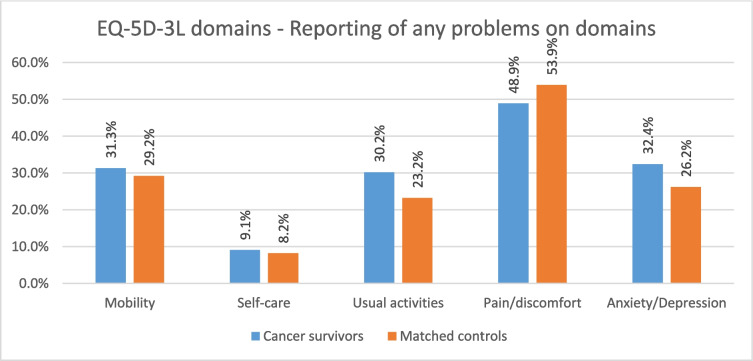
Comparison of cancer survivors and matched controls on EQ-5D-3L domains

#### Utility scores and VAS

No statistically significant differences between the cancer survivor and control cohorts were found for the EQ-5D-3L utility scores or VAS scores.

#### Domain scores

Chi-square analyses assessed differences in the proportion of cancer survivors and matched controls reporting any level of problem across the domains of the EQ-5D-3L and showed no statistically significant differences between groups on *mobility*, *pain* or *self-care*. However, a higher proportion of participants in the cancer survivor group reported problems with *usual activities* (30.2% vs 23.2%, *X*^2^ = 12.69, *p* < 0.001) and *anxiety/depression* (32.4% vs 26.2%, *X*^2^ = 9.29, *p* = 0.002) than those in the matched control group.

The colorectal cancer group reported significantly more problems with *usual activities* than their matched controls (32.1% vs 21.7%, *p* < 0.001). There were no other significant differences between the individual cancer groups and matched controls on any of the EQ-5D-3L domains. The comparisons are illustrated in the Supplementary file/Fig. 3.

### Cancer survivor identity (Supplementary file/Fig. 4)

There was a significant difference (*X*^2^ = 50.84, *p* < 0.001) between the cancer survivor group and the matched controls on the cancer survivor identity question (how they chose to describe someone who has had cancer). A higher number of participants in the cancer survivor group selected the option ‘A person who has had cancer’ than in the matched control group (55.9% versus 46.1%). A higher number of participants in the matched control group selected ‘A victim of cancer’ than in the cancer survivor group (8.2% versus 1.5%).

Differences between the breast cancer survivors and matched controls (*X*^2^ = 32.131, *p* < 0.001) and the colorectal survivors and matched controls (*X*^2^ = 27.882, *p* < 0.001) were significant, but not between the ovarian cancer group and matched controls. Responses across all subgroups had a similar pattern with cancer survivors being more likely to describe themselves as ‘A person who has had cancer’ and less likely to describe themselves as ‘A victim of cancer’.

### Healthcare usage and personal finances

#### Cancer Care Co-ordination Questionnaire (CCCQ)

Comparing the cancer survivors and matched controls on the navigation subscale of the CCCQ found no significant differences between any of the cancer survivor groups and their matched controls.

#### Healthcare usage

In terms of services used in the past 3 months, there were no statistically significant differences in the proportion of cancer survivors and matched controls who reported seeing their GP or ‘other’ healthcare providers, seeing a healthcare professional for emotional issues or problems with alcohol or drugs or accessing hospice care. A higher proportion of matched control participants reported seeing a physiotherapist (12.0% vs 8.3%, *p* = 0.009). No statistically significant differences between individual cancer survivor groups were observed when compared to matched controls.

Cancer survivors reported different patterns of hospital-based healthcare, being more likely than matched controls to require hospital care in the past 3 months (51.1% vs 44.1%) and more likely to access most of their care outside LTHT (*p* < 0.001). Differences between the breast survivors and matched controls were statistically significant in this regard (*p* < 0.001), as were differences between the ovarian survivors and matched controls (*p* = 0.003), with similar patterns observed. There were no differences between the colorectal survivors and matched controls.

A significantly higher proportion of cancer survivors reported accessing some type of voluntary or charity services (6.5% vs 3.8%, *p* < 0.001). However, the descriptions of services accessed were very heterogeneous and not always related to cancer.

#### Providing and receiving care

The matched control group was significantly more likely than the cancer survivor group to report providing care for someone else in the past 3 months (36.4% vs 29.9%, *p* = 0.002). These differences were significant between the breast survivor and control groups (*p* < 0.001) and the colorectal survivor and control groups (*p* = 0.004). However, there were no significant differences between the ovarian survivors and the control group.

Cancer survivors were more likely than their matched controls to report receiving care from someone else in the past 3 months (25.1% vs 18.9%, *p* < 0.001). These differences were significant between the colorectal survivors and their control group (*p* < 0.001) but not between the breast and ovarian survivors and their control groups.

There were no significant differences reported in the mean hours of care support received, the proportion of carers taking time off work and the mean number of hours that carers took off work.

#### Employment and income (Supplementary file/Table 5)

Statistical comparisons of employment status were not possible due to the small sample size, since the majority of participants in both groups were retired (61.4% of cancer survivors and 64.0% of matched controls). Reported income losses over the past 3 months were similar across both groups.

#### Medication, travel and other costs

Cancer survivors were less likely than matched controls to pay for their prescription medications (7.6% vs 17.9%, *p* < 0.001). However, there were no significant differences between the groups on costs of health and social care-related travel and parking or time spent travelling. The matched control group reported a higher expenditure on ‘other’ health and related costs than the cancer survivor group (£65 vs £37, *p* = 0.002). However, the description and amount of costs were very heterogeneous and included some descriptions that were not specifically cancer related (e.g. dentistry or house renovations).

## Discussion

This study offers novel insight into the impact of cancer 5 years after diagnosis for a cohort of cancer survivors in the UK by comparing outcomes on QoL, health, identity, healthcare usage and finances, with a cohort of control participants matched on age, sex and IMD. Results indicate that although the cancer survivor group had poorer QoL than matched controls as assessed by our primary outcomes (QLACS summary score), this difference was small. For specific domains, cancer survivors reported greater morbidity related to *energy/fatigue*, *finances*, *distress about family cancer*, *concerns about appearance*, s*exual interest/function* and *social avoidance*. There were no significant differences across *cognitive problems, positive feelings*, *negative feelings* or *pain*.

We compared the QLACS cancer-specific scores of the individual cancer groups (breast, colorectal and ovarian) and found that the breast group reported significantly worse QoL than the colorectal group. This is likely due to the younger age of the breast group, who may perceive worse QoL relatively to others in a similar age range, particularly if they have had more invasive treatment such as chemotherapy [11]. Previous research has suggested that about a third of colorectal cancer survivors do not return to their pre-treatment levels of QoL 5 years following surgery [7]. However, predictors of worse quality of life include non-cancer-related factors such as age and comorbidities, which may influence how colorectal survivors perceive their own QoL relative to their peers.

We found small but significant differences on the symptom subscales *tingling and numbness*, *urinary symptoms*, *diarrhoea* and *abdominal/GI symptoms*, but no differences on *muscular pain*, *urinary frequency*, *urinary incontinence*, *GI symptoms* or *constipation*. In subgroup analysis, we found that the breast group only differed significantly from controls on *tingling and numbness*, likely due to the use of adjuvant chemotherapy with taxanes. As would have been predicted, the colorectal group differed significantly on *diarrhoea* and *abdominal/GI symptoms*, but, surprisingly, also on *urinary symptoms*. This may be due to confusion with the wording of the item which asks ‘Have you had difficulty going out of the house because you needed to be close to a toilet’.

We found no differences between cancer survivors and controls on overall EQ-5D-3L utility or VAS scores but small significant differences on the proportion of participants reporting issues with usual activities and anxiety/depression, with the cancer survivors reporting more issues.

The Cancer Quality of Life Survey, a national project delivered by NHS England and NHS Digital, also gathered data from a range of cancer survivors approximately 18 months after diagnosis (https://www.cancerdata.nhs.uk/cancerqol), including EQ-5D-3L domain issues. In comparison to our data collected 5 years after diagnosis, a higher proportion of cancer survivors were reporting issues across all domains, indicating that overall, cancer survivor’s health improved in the time period between 18 months and 5 + years post-diagnosis, with the biggest improvements seen in *usual activities* and *anxiety/depression*.

The cancer survivor identity question illustrated that the majority of cancer survivors identified themselves as either ‘a cancer survivor’ or simply ‘a person who has had cancer’. Cancer survivors were less likely to identify with being a ‘victim of cancer’, compared to perceptions of the control group in how they would describe someone who has had cancer. Identifying as a ‘victim of cancer’ has been found to be associated with poorer general well-being [28], and the small proportions of cancer survivors identifying this way in our sample supports the general findings of relatively comparable QoL to the matched controls.

Across healthcare usage and finances, broadly, there were very few differences between the cancer survivors and matched controls who reported similar ease of navigating healthcare, healthcare usage and expenditure as well as similar levels of employment and income loss (albeit with the majority of the sample being retired). There were some differences, however, with the cancer survivor group being more likely to have accessed hospital care in the last 3 months and more likely to have had hospital care outside of the local NHS hospital. This may be accounted for by cancer-related aftercare in those diagnosed previously with cancer. This aftercare would be at the regional/specialist cancer centre which may not be their local NHS hospital, whereas the control cohort would have been referred to their local hospital as part of a 2WW for suspected cancer.

Cancer survivors were more likely than matched controls to be receiving care support from someone in the last 3 months and less likely to be providing care for someone else. However, there were no differences in the hours of support received, the proportion of carers taking time off work and the mean number of hours that carers took off work.

### Strengths and limitations

The main strengths of the study are the large sample, comprehensive range of outcomes assessed directly from participants and the timeframe since diagnosis, thereby providing a good representation of longer-term outcomes for cancer survivors following completion of the acute treatment phase. The matched control aspect of the study design offers novel insight into how some of the decline in QoL observed in non-matched studies may actually be attributable to non-cancer-related factors such as age and comorbidities.

However, there are some limitations. The response rate was low across both cancer survivors and matched controls, limiting the representativeness of both samples, particularly given the lower participation rates in the more socially deprived groups. It may well be that those with poorer health and QoL were less likely to participate.

Importantly, a significant limitation is the fact that we had limited demographic and clinical data available for participants, including age, sex and IMD but without detailed cancer diagnosis, stage and treatment. This work was part of a broader project [17], which aimed to create a ‘comprehensive patient record’ by linking de-identified data from primary care with hospital records in secondary care, and then add the PROMs data. Despite the benefits and advantages of data linkage, privacy issues remained a significant area of concern. The stakeholders involved in this project wanted to ensure that the method, its approaches and implementation were compatible with legal and ethical best practices at the time. Input was sought from external experts at the Confidential Advisory Group, NHS Digital (now NHS England), barristers, senior University leadership and many others. Delays due to the due diligence undertaken along with evolving work pressures on primary care data providers (in part due to COVID-19) and changes in the healthcare and data linkage landscape prevented the release and thus inclusion of the primary care data and enabled only a very limited linkage of hospital data to collect demographic variables.

The NHS Long Term Plan outlines the potential for integrating (PROMs) as part of improving care [31]. Theoretically, this data could be easily integrated with clinical and demographic data from EPRs and cancer registries to provide large datasets of good quality data [32–35], identify unmet needs and drive research and policy.

## Conclusion

Five years or more after diagnosis, the QoL of individuals living with and beyond breast, colorectal and ovarian cancer was generally similar to that of age, sex and IMD-matched controls. This finding has important implications for people affected by cancer and those providing care who would benefit from greater information on outcomes and functioning beyond treatment. Despite this reassuring finding, it is important to note that there were some differences, on both physical and psychosocial issues, mandating the need for specialist service provision.

## Supplementary Information

Below is the link to the electronic supplementary material.

Supplementary file1 (DOCX 65 KB)

## Data Availability

No datasets were generated or analysed during the current study.
