# Supplementary material for: Quality of life, healthcare usage and finances of UK cancer survivors five years post-diagnosis: a matched controlled study
Source: J Cancer Surviv. 2024 Dec 3;20(3):1035–45. doi: 10.1007/s11764-024-01708-x (PMC13144260; doi:10.1007/s11764-024-01708-x)
Supplement: Supplementary file 1 — Supplementary file1 (DOCX 65 KB) [file 11764_2024_1708_MOESM1_ESM.docx]

**Appendix/Supplementary File 1**

Table 4 QLACS domain comparisons and EORTC symptom comparisons for individual cancer groups and matched controls

|  | **Breast survivors** | | | **Matched controls** | | | **Mann Whit U** | **Colorectal survivors** | | | **Matched controls** | | | **Mann Whit U** | **Ovarian survivors** | | | **Matched controls** | | | **Mann Whit U** |
| --- | --- | --- | --- | --- | --- | --- | --- | --- | --- | --- | --- | --- | --- | --- | --- | --- | --- | --- | --- | --- | --- |
| **QLACS domains** | **N** | **Mn (SD)** | **Med (IQR)** | **N** | **Mn (SD)** | **Med (IQR)** | ***p*** | **N** | **Mn (SD)** | **Med (IQR)** | **N** | **Mn (SD)** | **Med (IQR)** | ***p*** | **N** | **Mn (SD)** | **Med (IQR)** | **N** | **Mn (SD)** | **Med (IQR)** | ***p*** |
| **Energy/Fatigue** | 412 | 12.9 (5.5) | 13.0 (8.0) | 401 | 11.9 (5.4) | 11.0 (7.0) | 0.004* | 367 | 12.0 (5.4) | 12.0 (7.0) | 364 | 11.5 (5.1) | 11.0 (6.0) | 0.173 | 57 | 12.8 (5.1) | 13.0 (7.8) | 55 | 12.2 (5.3) | 11.0 (6.0) | 0.512 |
| **Cognitive Problems** | 413 | 9.7 (4.8) | 9.0 (6.0) | 402 | 9.2 (4.8) | 8.0 (5.0) | 0.264 | 371 | 8.9 (4.4) | 8 (5.2) | 366 | 9.0 (4.4) | 8.0 (5.0) | 0.787 | 58 | 9.4 (4.7) | 8.5  (8.0) | 55 | 9.5 (4.2) | 9.0 (6.0) | 0.524 |
| **Positive Feelings** | 411 | 20.3 (5.5) | 21.0 (9.0) | 402 | 21.0 (5.3) | 22.0 (8.0) | 0.035 | 367 | 21.2 (6.0) | 23.0 (10.0) | 364 | 21.2 (5.5) | 22.0 (9.0) | 0.951 | 58 | 20.4 (6.0) | 23.0 (9.8) | 55 | 20.5 (5.1) | 21.0 (7.0) | 0.780 |
| **Negative Feelings** | 411 | 11.0 (5.1) | 10.0 (7.0) | 402 | 10.6 (5.1) | 9.0 (6.0) | 0.227 | 367 | 9.5 (4.4) | 8.0 (6.0) | 363 | 9.4 (4.5) | 8.0 (6.0) | 0.892 | 58 | 10.9 (5.3) | 10.0 (6.8) | 54 | 10.7 (4.9) | 9.0 (6.0) | 0.644 |
| **Financial Problems** | 413 | 7.4 (5.4) | 5.0 (5.0) | 400 | 5.0 (2.7) | 4.0 (0.0) | <0.001* | 372 | 6.3 (3.9) | 4.0 (3.0) | 369 | 5.1 (2.8) | 4.0 (0.0) | <0.001* | 58 | 6.6 (3.9) | 4.0 (5.0) | 56 | 4.7 (2.0) | 4.0 (0.0) | 0.001* |
| **Distress about family cancer** | 410 | 8.8 (5.4) | 7.0 (8.0) | 400 | 5.9 (3.7) | 5.0 (4.0) | <0.001* | 371 | 7.8 (4.8) | 7.0 (5.0) | 364 | 5.5 (3.5) | 4.0 (4.0) | <0.001* | 57 | 7.2 (4.4) | 6.0 (6.0) | 55 | 6.1 (4.6) | 4.0 (6.0) | 0.065 |
| **Appearance concerns** | 410 | 9.3 (6.1) | 7.0 (8.0) | 398 | 6.4 (4.6) | 4.0 (3.0) | <0.001* | 370 | 6.6 (4.2) | 4.0 (4.0) | 366 | 5.7 (3.6) | 4.0 (2.0) | <0.001* | 58 | 6.8 (4.2) | 5.2 (3.0) | 55 | 7.0 (4.6) | 4.0 (4.0) | 0.350 |
| **Sexual Interest Function** | 376 | 12.3 (6.7) | 12.0 (10.0) | 373 | 10.7 (5.8) | 10.0 (9.0) | 0.002* | 329 | 11.8 (6.2) | 11.0 (10.0) | 348 | 10.9 (6.2) | 10.0 (10.0) | 0.073 | 50 | 13.2 (6.4) | 13.5 (9.0) | 53 | 10.7 (5.4) | 10.0 (8.0) | 0.116 |
| **Pain** | 413 | 10.6 (6.1) | 9.0 (8.0) | 404 | 10.2 (5.9) | 8.0 (7.9) | 0.236 | 370 | 9.9 (5.6) | 8.0 (7.0) | 368 | 10.0 (6.0) | 8.0 (7.0) | 0.488 | 58 | 9.2 (5.2) | 7.5 (6.8) | 56 | 9.7 (5.8) | 8.0 (7.0) | 0.421 |
| **Social Avoidance** | 412 | 8.6 (5.5) | 7.0 (7.0) | 402 | 7.9 (5.2) | 6.0 (6.0) | 0.071 | 367 | 8.1 (5) | 6.8 (6.0) | 367 | 7.7 (5.0) | 6.0 (6.0) | 0.070 | 57 | 8.2 (4.8) | 7.5 (6.5) | 56 | 8.6 (6.0) | 7.0 (6.0) | 0.888 |
| **Distress about recurrence** | 411 | 13.2 (7.0) | 11.0 (11.0) | - | - | - | - | 374 | 10.7 (6) | 9.0 (7.5) | 0 |  |  | - | 56 | 15.3 (6.7) | 15.0 (10.0) | - |  |  |  |
| **Benefits** | 409 | 17.2 (6.7) | 18.0 (10.0) | - | - | - | - | 366 | 16.6 (6.5) | 16.0 (10.0) | 0 |  |  | - | 56 | 11.7 (6.0) | 10.5 (6.5) | - |  |  |  |
| **EORTC symptom subscales** | **N** | **Mn (SD)** | **Med (IQR)** | **N** | **Mn (SD)** | **Med**  **(IQR)** | ***p*** | **N** | **Mn (SD)** | **Med (IQR)** | **N** | **Mn (SD)** | **Med**  **(IQR)** | ***P*** | **N** | **Mn (SD)** | **Med (IQR)** | **N** | **Mn (SD)** | **Med** | ***P*** |
| **Tingling or numbness** | 408 | 18.7 (27.1) | .00 (33.3) | 396 | 15.3 (26.2) | .00 (33.3) | 0.009 | 363 | 18.5 (29.1) | .00 (33.3) | 361 | 17.6 (27.8) | .00 (33.3) | 0.017 | 56 | 22.6 (28.3) | .00 (33.3) | 55 | 14.8 (25.6) | .00 (33.3) | 0.099 |
| **Muscular pain** | 406 | 46.3 (28.9) | 33.3 (33.3) | 403 | 43.5 (29.5) | 33.3 (33.3) | 0.162 | 364 | 41.8 (28.5) | 33.3 (33.3) | 366 | 42.3 (29.4) | 33.3 (33.3) | 0.774 | 57 | 35.2 (33.0) | 33.3 (66.6) | 56 | 44.4 (32.4) | 33.3 (33.3) | 0.310 |
| **Urinary frequency** | 412 | 36.7 (27.9) | 33.3 (33.3) | 403 | 35.6 (25.6) | 33.3 (33.3) | 0.444 | 370 | 39.9 (27.3) | 33.3 (37.5) | 366 | 35.7 (24.8) | 33.3 (33.3) | 0.357 | 58 | 30.2 (21.4) | 33.3 (33.3) | 56 | 34.3 (20.8) | 33.3 (33.3) | 0.428 |
| **Urinary incontinence** | 402 | 17.1 (26.0) | .00 (33.3) | 395 | 17.0 (25.1) | .00 (33.3) | 0.745 | 358 | 17.0 (26.6) | .00 (33.3) | 362 | 12.3 (20.2) | .00 (33.3) | 0.059 | 57 | 12.0 (20.8) | .00 (33.3) | 56 | 14.8 (19.1) | .00 (33.3) | 0.275 |
| **Urinary symptoms** | 404 | 8.4 (21.3) | 0 | 395 | 6.8 (18.8) | 0 | 0.331 | 360 | 13.9 (25.3) | .00 (33.3) | 364 | 5.3 (19.6) | .00 (.00) | <0.001* | 56 | 5.0 (12.0) | .00 (.00) | 56 | 8.6 (20.7) | .00 (.00) | 0.711 |
| **GI symptoms** | 409 | 19.2 (27.1) | .00 (33.3) | 402 | 19.9 (25.7) | .00 (33.3) | 0.274 | 369 | 14.9 (23.2) | .00 (33.3) | 365 | 18.1 (25.9) | .00 (33.3) | 0.256 | 58 | 12.0 (22.7) | .00 (33.3) | 56 | 20.4 (24.6) | .00 (33.3) | 0.057 |
| **Diarrhoea** | 407 | 10.9 (22.2) | .00 (00) | 400 | 9.4 (19.4) | .00 (.00) | 0.474 | 360 | 19.4 (27.7) | .00 (33.3) | 364 | 10.4 (20.0) | .00 (.00) | <0.001* | 57 | 8.2 (17.2) | .00 (.00) | 55 | 12.3 (24.5) | .00 (8.33) | 0.467 |
| **Constipation** | 412 | 19.1 (28.9) | .00 (33.3) | 403 | 16.0 (25.5) | .00 (33.3) | 0.485 | 366 | 16.9  (25.5) | .00 (33.3) | 363 | 14.5 (24.5) | .00 (33.3) | 0.069 | 57 | 23.9 (28.8) | 33.3 (33.3) | 56 | 14.2 (23.9) | .00 (33.3) | 0.063 |
| **Abdominal/GI symptoms** | 411 | 16.1 (18.2) | 13.3 (26.6) | 403 | 15.4 (17.1) | 13.3 (26.6) | 0.852 | 364 | 17.6 (19.1) | 13.3 (26.6) | 366 | 13.0 (16.0) | 6.6 (20.0) | <0.001* | 58 | 15.5 (15.8) | 6.66 (26.6) | 56 | 13.6 (15.5) | 6.66 (20.0) | 0.662 |

Figure 3 Comparison of individual cancer groups and specific matched control groups on presence/absence of problems on EQ5D-3L

Figure 4 Comparison of cancer groups and matched controls on cancer survivor identity question

Table 5 Comparison of employment between cancer survivors and controls

|  | **Cancer survivors** | | **Matched controls** | |
| --- | --- | --- | --- | --- |
|  | **N** | **%** | **N** | **%** |
| **Full time employment (more than 30 hours/week)** | 130 | 15.6% | 187 | 15.7% |
| **Part time employment (less than 30 hours/week)** | 102 | 12.3% | 130 | 10.9% |
| **Self employed** | 26 | 3.1% | 39 | 3.3% |
| **Student or training scheme** | 3 | 0.4% | 3 | 0.3% |
| **Retired** | 510 | 61.4% | 760 | 64.0% |
| **Unemployed, seeking work** | 7 | 0.8% | 6 | 0.5% |
| **Unemployed, unable to work for health reasons** | 31 | 3.7% | 36 | 3.0% |
| **Not in paid employment due to looking after family/home** | 7 | 0.8% | 12 | 1.0% |
| **Other** | 15 | 1.8% | 15 | 1.3% |
